# The indignities of shielding during the COVID-19 pandemic for people with sickle cell disorders: an interpretative phenomenological analysis

**DOI:** 10.3389/fsoc.2024.1334633

**Published:** 2024-02-13

**Authors:** Maria Berghs, Francesca Horne, Scott Yates, Rachel Kemp, Amy Webster

**Affiliations:** ^1^Allied Health Sciences, De Montfort University, Leicester, United Kingdom; ^2^Nottingham Trent University, Nottingham, United Kingdom; ^3^Applied Social Sciences, De Montfort University, Leicester, United Kingdom; ^4^Purple House, Rugby, United Kingdom; ^5^University Hospitals of Leicester NHS Trust, Leicester, United Kingdom

**Keywords:** shielding, COVID-19, sickle cell disease, pandemic, ableism and racism

## Abstract

This article seeks to understand the first-hand experiences of people with sickle cell, a recessively inherited blood disorder, who were identified as clinically extremely vulnerable during the COVID-19 pandemic. Part of a larger sequential mixed-methods study, this article uses a selective sample of eight qualitative semi-structured interviews, which were analysed using interpretative phenomenological analysis (IPA). The first stage of IPA focused on practical concerns participants had correlated to understanding shielding and their feelings about being identified as clinically extremely vulnerable. In a secondary stage of analysis, we examined the emotions that it brought forth and the foundations of those based on discriminations. This article adds to our theoretical understanding of embodiment and temporality with respect to chronicity and early ageing. It explains how people with sickle cell disorders have an embodied ethics of crisis and expertise. It also elucidates how people’s experiences during the pandemic cannot be seen in void but illustrates ableism, racism, and ageism in society writ large.

## Introduction

On 23 March 2020, a national ‘lockdown’ was announced in the United Kingdom by the then British Prime Minister, Boris Johnson, in response to the threat from the severe acute respiratory syndrome coronavirus (SARS-CoV-2), now commonly known as COVID-19. While different in each of the devolved nations, in England, ‘lockdown’ was typified by the public being asked to ‘stay at home’, limit social contacts, ensure social distancing, and only leave the house for essentials, such as to go food shopping or attend urgent medical appointments. The lockdown was initially introduced for a short period of 12 weeks but lasted for about a year, ending officially on 19th of July 2021. People with compromised immune systems, serious illnesses, disabilities, or chronic conditions were identified, initially by the National Health Service (NHS) and then by their GPs, as ‘clinically extremely vulnerable’ (CEV) to the COVID-19 virus and were asked to ‘shield’ by the government. Shielding was a novel societal concept, defined as ‘extreme isolation’ with the aim of protecting people classified as CEV or thought to be at high risk, such as older people.

The main rules for people who were CEV included: (1) not leaving a household unless urgently required, such as for medical reasons and (2) isolating from other members of the household. To aid people in shielding, many initiatives were introduced, such as the provision of food parcels, virtual GP and consultant appointments, medication deliveries, and working from home policies. While there were provisions made to ensure social contacts, such as allowing families and singles to form ‘bubbles’ with other households, people who were shielding were initially excluded from such measures. Being in bubbles also did not protect people from the effects of ‘extreme isolation’, including increased loneliness as well as anxiety and depression (Carr et al., 2021; Fancourt et al., 2021). For instance, evidence from the English Longitudinal Study of Aging (Di Gessa and Price, 2022) shows that older adults (50+) who were shielding experienced worse mental health.

Despite concerns about the physical and mental health of CEV people who were shielding, a complex picture was emerging. Kemp et al.’s (2020) study in the early days of shielding found that high-risk patients felt vulnerable and anxious but also that they were very resilient in coping. Westcott et al.’s (2021) study with cystic fibrosis patients noted that depression rates were low and remained stable, while there were higher rates of anxiety. Similarly, while they had a small sample (*n* = 25), Kemp et al. (2020) also found that almost half of the patients they surveyed felt that shielding had not really changed their lives, as due to their condition of multiple myeloma, they were already living in social isolation. Robinson et al. (2022), in a systematic review of longitudinal studies, found that while mental health was affected early in the pandemic from March 2020, it decreased mid-pandemic and was comparable to pre-pandemic levels in most population groups.

These studies show nuances in how people experienced shielding but do not investigate the underlying causes of poorer mental health. In this article, we try to understand some of these underlying causes by reporting on qualitative data from a sequentially designed mixed-methods study conducted during the pandemic. We document physical and mental health in a small cohort of participants of African and African Caribbean origin who had sickle cell disease (SCD).

In England, SCD is one of the most common genetic conditions, with estimates of 14,000 people affected (Dormandy et al., 2018). The condition affects the haemoglobin in red blood cells, causing them to form sickle-like shapes, which can cause blockages to the major organs and severe pain, necessitating emergency care, which is called a crisis (Kavanagh et al., 2022). The causes of a pain ‘crisis’ can be exacerbated by a host of different factors, including (1) environment, such as extremes in temperature or strenuous exercise; (2) physiological, like a lack of hydration or proper nutrition; (3) psychological, in terms of stress or excitement; and (4) structural issues correlated to inequalities, such as the inability to access proper housing or good healthcare (Dyson, 2011).

SCD is thus characterised by its uncertainty (Rouse, 2009), in that while there is a severe (HbSS) and mild (HbSC) version, depending on varied circumstances affecting a person, it can be a chronic condition, in others it is a disability, and for some it is life-threatening, but it can also be all these things for one and the same person. As people age with SCD, they will also experience more complications linked to SCD as well as co-morbidities and impairments due to ageing and this will happen at an earlier and accelerated time than in the general population (Idris et al., 2022). Yet, in day-to-day life, most people with SCD, even if they are experiencing pain or fatigue, look fine, which is why SCD is viewed as an invisible condition or disability (Ciribassi and Patil, 2016) but is often bureaucratically contested as a disability (Das, 2022; Srikanthan, 2023). Within healthcare settings, where people with SCD encounter structural racism, identification with affirmative Black identity and explaining SCD as a serious medical condition often take precedence over any ‘disability’ identity. Even in schools and employment settings, the identification of disability identity and ascription of necessary rights could be a struggle for people who outwardly might look ‘able’ and/or have to deal with other discriminations (see Berghs and Dyson, 2022). The pandemic seemed to be the first time that there was this public bureaucratic and medical recognition of people with SCD as having a clinically serious condition.

We felt it was important to not only try to identify any physical or mental health needs in this population using a validated quality of life measure (SF36) (see Berghs et al., 2022) but also to deploy qualitative, semi-structured interviews to understand how the phenomenon of shielding and identification as a CEV person was experienced. Furthermore, using an interpretative phenomenological analysis (IPA), we explored the emotions that this brought forth and found that participants felt that discriminations within society were heightened during the pandemic. Even though some participants reported health improving, most participants reported worse mental health as the pandemic raised risks correlated to how SCD is embodied, temporality with respect to chronicity, and understandings of ageing and mortality.

### Background

Ableism, disablism, ageism, and racism are all forms of discriminations (Overall, 2006) that became foregrounded during the global pandemic. Lockdown was a divisive policy in the popular press and amongst scientists, with the health of a minority ‘vulnerable’ to COVID-19 pitted against the economic health of the state and the majority who were fit and healthy (Dorling, 2020). Inclusion London (2020) noted how disabled people felt forgotten and ignored during the pandemic despite policies of lockdown. Thus, Arcieri (2022) found that increased anxiety amongst older adults during the pandemic was correlated to ageism and ableism in society. Ableism (Campbell, 2008, 2009), the prioritisation of fit and healthy bodies (Andrews et al., 2021), and disablism, the discrimination against people with impairments causing them to become ‘disabled’ (Oliver, 1983), such as premature deaths of people with intellectual disabilities, were defining features of the pandemic (Walmsley, 2020; Heslop et al., 2021; Chicoine et al., 2022).

The pandemic also exposed how being identified with ‘pre-existing conditions’ in clinical settings could bring up discussions of ‘quality of life’ and curtail rights to life-saving treatments (Abrams and Abbott, 2020). The medical rationing of ventilators, triaging with limited access to treatment, and negative psychological impact of shielding language and practices on older populations in the early pandemic highlighted ageism in society (Monahan et al., 2020; Ramirez et al., 2022). Ableism was also foregrounded in how previously inaccessible hybrid and flexible working policies, as well as reasonable accommodations and equipment to be able to work from home, were granted during the pandemic (Ocean, 2021; Samuels and Freeman, 2021). Masking mandates and the refusal of some people to wear masks to protect populations made ‘vulnerable’ were also deemed ableist (Grunawalt, 2021).

Older and disabled people also argued that the way in which their health and social care was organised, for example, in care homes, by support workers, or in hospitals, did not allow for shielding, necessitated embodied contact, and left them more vulnerable to the risks of COVID-19 infection and death (see Shakespeare et al., 2021). This also indicated unequal class and other privileges in society, with some population groups able to shield, maintain social distancing, stay at home, and mask, while others could not shield or had to work, for example, as frontline healthcare workers. Goodley et al. (2023) note that this also heightened the feelings of vulnerability in the chronic and disabled populations and brought to the fore anxieties and fears. Yet, they also note how the pandemic also brought with it affirmation of life and resilience in mutual aid groups, peer interventions, and adaptation (Goodley et al., 2023). Samuels and Freeman (2021) found that the pandemic highlighted ‘crip-time’ in that disabled and chronically ill people often experience time differently from the ‘chrononormative’ and ableist way that time is used to structure and control life. They found that ‘crip-time’ (Kafer, 2013, 2021) was what everyone began to experience during the pandemic; in that time was no longer linear but unpredictable. They argue that while crip-time can be liberating, it can also feel enforced as it becomes predicated on the disabled body–mind and how temporality is interlinked to ‘race’, colonialism, gender and sexuality’ (Samuels and Freeman, 2021).

Goodley et al. (2023) and Samuels and Freeman (2021) thus mostly highlight an elitist positioning in resistance to ‘slow time’, noting how some disabled people are not granted any ‘time’ nor ‘survival’. Furthermore, Levy et al. (2023) argue that the pandemic acerbated a crisis of ageism in how policies and language used during the pandemic created such vulnerabilities, which affected some people over others. The way in which the pandemic was spoken about was often in militaristic terminology; for instance, as an invisible enemy, people had to ‘fight’ with healthcare workers viewed as self-sacrificing individual ‘heroes’ (see Cox, 2020). The militaristic language also brought to the fore the able-bodied warrior as the patriarchal ideal productive norm of late capitalist society. Tremain (2023) argues that this is ‘disaster ableism’ in how the pandemic could be exploited to normalise the norms, values, and beliefs of ableism. The militarised language hid the fact that it was inequalities and structural violence in a lack of welfare provision that made certain population groups more vulnerable, which was in turn, caused by government neoliberal economic policies (Lohmeyer and Taylor, 2021). Barrett et al. (2021) found that the language of sacrifice on Twitter, during the pandemic indicated a ‘calculated ageism’ in that the older population was deemed worthy of sacrifice for the younger generation and the economic needs of a country.

Thorneycroft and Asquith (2021) thus theorise that the pandemic brought to the fore and made visible fears of societal and structural ‘violence’ in becoming disabled, abject, and thus disposable. Like other scholars, they argue that the pandemic represented a necropolitical (Mbembe, 2003) continuum of neoliberal capitalism, where the state becomes sovereign in deciding which bodies matter and which do not (Thorneycroft and Asquith, 2021). This necropolitical order was also underpinned by class and racial inequalities, which were made more visible during the pandemic in the deaths of minoritised populations (Sandset, 2021; Ramirez et al., 2022), as well as amongst frontline healthcare workers. Early in the pandemic, Laster Pirtle (2020) contended that even in a supposedly ‘deracialised’ neoliberalism, COVID-19 was another feature of racialised capitalism, noting that it was racialised and economically deprived groups that would be impacted the most. These concerns became highlighted with the death of George Floyd on 25 May 2020 and the Black Lives Matter movement during the pandemic (Sobo et al., 2020). Black people have always suffered unequal violence in structural discriminations in embodied distress, chronicity, disability, illness, and deaths, but the pandemic made these disparities more visible (see Carney et al., 2023). The embodied impacts of inequalities, stress, and discriminations that lead to adverse life outcomes, bad physical and mental health, as well as accelerated ageing and disability, have furthermore been linked to bodies ‘weathering’ such traumas and accelerated ageing (Geronimus, 2023).

While ableism, racism, and disablism can exist together, they must also be seen as separate from each other. Adopting an intersectional lens (Crenshaw, 1991) means that we can differentiate between (1) ageing with physical, cognitive, sensory, and emotional impairments, (2) gaining impairments as we age, and (3) discriminations, such as becoming ‘disabled’ which, according to a social model of disability, is when society discriminates against people with impairments (Oliver, 2013). Similarly, van der Horst and Vickerstaff (2022), using Thomas’s (1999) social-relational model of disability, describe how, for them, ageism is a form of social oppression. Thomas (2012, 2019) argued that disablism allowed disability to come to the fore through avoidable discriminatory practices and oppressions. She also made a distinction between ‘restrictions of activities’ that were not disabilities. Instead, Thomas (2012, 2019) argued that impairments and impairment effects (the direct and unavoidable impacts of impairments on embodied functioning), were bio-socially and culturally constructed, in that while they may be predicated on biology or the body, they were also socially and culturally created too. Hence, van der Horst and Vickerstaff, (2022) argue, “Ageism would be differential treatment based on age, not based on impairments. Differential treatment based on (real or expected) impairments would be ableism.” Ageism then becomes separated from the effects of ageing or ‘age effects’, which may be different from ‘impairment effects’ and ableism.

In the pandemic, discriminations and oppressions worsened, and it seemed as if they could have an impact on age and impairment effects. Yet, the evidence from the pandemic also seems to indicate that we view age and impairment effects as monolithic when people can have multiple age and impairment effects. Similarly, age effects, like co-morbidities, might worsen because of worsening impairment effects. We also note how evidence seems to suggest that discriminations can accelerate ageing and impairments. This also calls into question ageing as visible and temporally constructed to a norm, as there may be forms of disability that are correlated to early ageing. In what follows, we try to contextualise the above literature and theory to the experiences of people with SCD as CEV and shielding during the pandemic.

## Methods

The study was conducted in the Midlands region of England between June 2020 and June 2021, during the COVID-19 pandemic. The Midlands region encompasses the city of Birmingham, which had some of the highest rates of COVID-19 deaths in NHS Trusts outside of London, as well as having some of the longest periods of lockdown with the city of Leicester (see Berghs et al., 2022). Ethical approvals for this project were given by the De Montfort University Faculty of Health and Allied Health Sciences Ethics Committee in 2020. The project was co-produced with two voluntary organisations based in the Midlands, OSCAR Birmingham and OSCAR Sandwell, who were also responsible for sharing information about the project.

We used a sequential mixed-method design to explore the perspectives of people with SCD towards shielding during the COVID-19 pandemic (Berghs et al., 2022). The qualitative phase of the study used interpretative phenomenological analysis (IPA) due to the methodological need to understand the phenomenon of shielding from participants’ perspectives (Smith and Fieldsend, 2021). IPA has been used successfully in previous SCD psychology research using smaller sample sizes (Erskine, 2012; Coleman et al., 2016). Semi-structured interviews were conducted at different points throughout the pandemic, with one being conducted after the end of shielding to ensure comparison. All interviews lasted around 50 min to an hour and a half and were audio-recorded, with verbal and written consent being given by all participants (Bryman, 2016; Lobe et al., 2020). We also ensured that a Black person with SCD who had a background in counselling was available for interviews and that we could signpost participants to the voluntary sector for further support if needed.

We felt this was ethically important due to the sensitive issues that came to the fore around racism and deaths in NHS services. For the same reasons and increasing pressures on the voluntary sector as the pandemic progressed, we decided to switch to IPA, allowing us to recruit a smaller sample size (6–8 people), but that would allow a deeper understanding of a phenomenon from the participant’s perspective (Smith and Fieldsend, 2021). In total, eight participants with SCD took part in the interviews, comprising six women and two men. The in-depth, semi-structured interviews occurred via the online platforms of WhatsApp, Zoom, and Teams and were audio-recorded (Lobe et al., 2020). Participants were selectively sampled (Bryman, 2016) to determine whether their physical health deteriorated, improved, or remained the same during the pandemic. We also chose to recruit an even split of people with SCD who had caring responsibilities and people who did not, as well as those employed and unemployed, although one person was experiencing furlough and another was having a break from work. Three participants were in their 20s, three participants were in their 30s, one participant was in their 40s, and one was in their 60s.

We used the qualitative software analysis programme NVivo to organise and code our data according to a dual hermeneutics, which ensured we stuck closely to participants’ understandings (Pietkiewicz and Smith, 2014). The emergent themes were also checked against any theories or findings correlated to shielding (Layder, 1998). We checked data analysis and thematic coding with a clinical psychologist and other members of the research team to ensure interpretation was sticking closely to how participants were making sense of their experiences of CEV and the phenomenon of shielding. We also triangulated the data with the findings from the survey to ensure further rigour, validity, and contextualisation (Bryman, 2016). Initially, we thus examined NHS treatment and care as those were significant codes and themes in our data analysis and triangulated strongly with the quantitative data (Berghs et al., 2022). However, to understand those experiences, we had to contextualise being identified as CEV and what shielding entailed for people. We initially examined emotions but realised that those became connected to experiences of racism and ableism. In this article, we report on those wider thematic findings from the semi-structured interviews using IPA.

## Findings

In our quantitative analysis, we found that there was worse quality of life and mental health during the pandemic, and this was linked to discrimination (Berghs et al., 2022). In the IPA of the qualitative data, we identified three themes that came out of people’s experiences of shielding that were also strongly correlated to understanding the intersectionality of those oppressions (Crenshaw, 1991). First, the indignities of the recognition of embodied vulnerabilities that had once been contested and invisible (Ciribassi and Patil, 2016; Srikanthan, 2023). Second, the importance of time and temporality to understand the context of the pandemic (Samuels and Freeman, 2021) for a chronic condition typified by uncertainty and early ageing (Sheppard, 2020). Finally, chronicity brought into context temporality and fears of mortality.

### Indignities of recognition: embodiment and vulnerabilities

Despite SCD being the most common inherited genetic condition in England, it has been historically neglected and misunderstood by healthcare professionals as well as members of the public, pointing to entrenched inequalities in terms of structural racism (Dyson, 2011). With the introduction of shielding, it became visible, and participant 2 (female, 20s) stated, “I feel like it was the first time that sickle cell was actually recognized by the government as a serious condition.” Recognition was a double-edged sword; as participant 1 (female, 30s) pointed out, it was because COVID-19 could ‘kill’ them. SCD was mentioned on government websites, people were being contacted by the government or their GPs, and some were able to receive charitable aid, such as food parcels.

As noted by Shakespeare et al. (2021), recognition was also felt to be superficial in nature, as some participants initially reported not being able to get information about shielding rules or not being contacted by GPs, consultants, or SCD centres at hospitals. Participant 4 (female, 30s) explains, “And I was petrified, And then I did not have no one to turn to.” Participant 2 wondered: if they were so vulnerable and at risk, why could they not have free prescriptions? Additionally, the food parcels ignored their cultural and religious backgrounds, as well as impairment and age effects impacting on activities like being able to open canned goods or even cook. Participant 3 (male, 60s) stated, “I mean, I’m in the survival mode.” And he also reminded the interviewer of the uncertainty of impairment effects in that, “We have a time where we cannot even stand up.”

Some participants, while not naming it, mentioned ableism (Campbell, 2008, 2009), for example, how flexible working from home had not been an option but was now possible for their employers and made accessible (Ocean, 2021; Samuels and Freeman, 2021). Participants noted that individual self-management of the risks was how they coped with shielding, especially during tiers 3 and 4 lockdowns, which were the highest levels. Participant 3 (male, 60s) explained:

“If I go to the shops, I've got disposable gloves. You know once I get in the car. I take them off, sanitize and then I will hold the steering wheel. I won't go through the shops, that is like a cattle market for some reason (…) you would never think there was a pandemic. So, during this, especially during this three-tier system I've driven to (town) to go to (supermarket) you're talking an hour, an hour, 40 minutes-drive one way, you're talking a five-and- a-half-hour journey. That's how I cope in my head.”

In this way, conceptions of time became interwoven with greater vulnerabilities and ‘survival’ as practical acts of ‘taking time’ in resistance and affirmation (Goodley et al., 2023). Despite the emphasis on individual self-management, there was also the gradual realisation that shielding would not be possible due to the kinds of embodied care they had to give, for instance, as mothers, and the relational support they depended on from others for their healthcare and other needs, as noted by Shakespeare et al. (2021). Participant 5 (male, 40s) stated:

“I have a support worker, here in the house. But she just comes like in the morning and goes shopping and does the dinners, but just the dinner, not lunch. So even, like they said to me to shield myself, like just stay in the room. I couldn't, I had to do my own shopping and go to the pharmacy to collect my medication and all that.”

He explains being really scared at the start of the pandemic because he was living with people who were not shielding. This was often contrasted by participants who were living with family members and had to adopt extreme measures to ensure shielding. Participant 7 (female, 30s) explained:

“So as soon as they used to finish work, when they, when they get home, they will change up by the door, strip right off, put things into black bags, straight to the washing machine, going upstairs to go have a shower and just, you know, before even like talking to me, they've done all of that.”

Despite the lengths taken to ensure shielding, many participants also related how the rules had to be ‘broken’ to go into hospitals for regular blood transfusions, pain management, or emergencies. For example, participant 8 (female, 20s) noted shielding was an illusion:

“I had to leave my house during the shielding period, because I had to go into hospital and I remember thinking, oh, my gosh, I don't think I could go to the hospital because as soon as I step out the door it's going to just hit me, like this coronavirus is airborne and like, you get really scared and paranoid. And I remember feeling so scared.”

Shielding thus heightened feelings of vulnerability but also a sense that the authorities had not taken their condition seriously before nor provided the necessary health and social care services (Lohmeyer and Taylor, 2021). As participant 3 (male, 60s) stated:

“There’s a lot of contradiction, so on paper, I’m vulnerable (…) They’ll recognize that I’m vulnerable (…) But at the same time, immediate social services are not there.”

Participant 6 (female, 20s) also related feeling the same way:

“Me as an individual who is a single person, as part of the BAME community, who's got health conditions, who's classed as vulnerable and recognized as vulnerable by the government, by the system, is treated like I've got nothing.”

This meant that most of our participants felt different from other populations who were CEV because of the intersectional nature of their vulnerabilities. Participant 8 (female, 20s) brought up inequalities and the prioritisation of people who had cystic fibrosis or cancer and how they had funded services even during the pandemic. We noted that this feeling of neglect also became correlated to understandings of time in shielding and to how ill a person was. Participant 8 states:

“And because I felt like, I had to be going into hospital, and see doctors and stuff like that regularly. It kind of, it made me feel like I wasn't shielding in a way. That meant that, like I didn't feel like shielding in the same way. I felt like I was in a different kind of phase.”

### Temporality

As illustrated above, the importance of time and timing for pandemic preparedness and support (Shakespeare et al., 2021) came up a lot during the interviews to understand why shielding was not possible. Some of the participants brought up the suddenness at which shielding began and how it was different from social distancing, like participant 1 (female, 30s):

“It all happened really quickly. Really, I just thought I’ll be socially distancing like everybody else you know, then it turned into, actually you have to shield and you can’t even leave your house at all. So, I did not get much time to get my head around things, before that was what we were meant to be doing.”

However, participants 2 (female, 20s) and 6 (female, 20s) noted how people with SCD and their families live in anticipation of a crisis, explaining how their mothers prepared and stocked up on food and essentials. Many participants noted that for them, just to be ‘normal’ means precision timing and planning for any impairment effects (Thomas, 2012, 2019). Participant 1 (female, 30s) explains this well:

“You’d be like, “Okay, what should we do today?” It’s a bit chilly, so that means we can’t go here, we can’t go there, we’ve got to go somewhere inside, to live normally takes a level of military planning, like make sure I am wearing the right things, make sure I have my medication, making sure to keep hydrated, how long is the walk, can I park, is it steep, you know you’ve got to go through this almost like mental checklist anytime you go anywhere.”

Normal time outside of the pandemic, or ‘chrononormativity’ as mentioned by Samuels and Freeman (2021), entailed that people with SCD could physically not always have time to rest their bodies and look after themselves; thus, some participants reported having better health during the pandemic. Participant 7 (female, 30s) said that they were able to feel ‘well-rested’ and thus, “I think it’s better. I think it actually is better this year.” This was echoed by participant 4 (female, 30s):

“It was better this year. I would say it probably the best I've been as an adult. I don't think I've actually experienced a crisis. I think I had one earlier in the year. But what I've noticed is, with me not going out to work. I wasn't as tired, and my body wasn't as stressed out. And I think that actually helped to, like being able to work from home and I noticed the difference.”

Participants related that they also tried to avoid going into the NHS as they felt as if they were always being treated as if they were ‘wasting the time’ of healthcare workers, or as participant 5 (male, 40s) explained, being a ‘burden’. Pain management was thus mostly done at home, but there were participants who stated that their health got worse and they needed to go to the hospital.

With respect to ‘normal’ time (Samuels and Freeman, 2021), some participants related keeping to routines, especially if they had children and work to do. Like participant 4 (female, 30s), “I usually get up the normal time I’d get up to go to work, I kept that routine.” This was different for participants 2 (female, 20s) and 5 (male, 40s), who overturned norms of time. Participant 5 stated that this helped with his shielding. “At the moment it’s a bit upside down. Like, I sleep a lot in a day (…) it is in reverse. So, I sleep during the day in and go, like going around more at 2 am, 3am, 4 am I’m still awake.” All participants related that people with SCD needed to take time to sleep, rest, and slow down to manage their conditions, but also because they could forget things due to impairment effects (Thomas, 2012, 2019), like, fatigue. For instance, participant 5 noted that not having face-to-face consultations and check-ups was a problem, “on the phone it’s different because it’s so quick and sometimes you forget stuff.” The importance of taking time and living in each moment was also related by participants as important to resilience and affirmation of life (Goodley et al., 2023). Participant 3 (male, 60s) stated: “That’s how I cope with it, I just took it day by day. Do not look beyond that day, though. (…) As you know, Christians will say in the cultural background, tomorrow’s not promised to nobody.”

If every moment of time is precious, giving time to someone also shows their importance, and not giving time seemed to illustrate ableism and disablism in services and a lack of understanding of SCD. This was highlighted by participant 6 (female, 20s) observing triaging of patients: “I feel like COVID had an impact as well, I feel like they were so busy and stuff like that (…). And then try to tell you that you are not as important as this person.” The lack of improvement in NHS services over time and the fact that some people felt that they were treated worse during the pandemic meant that some participants felt that time was not moving forward. Participant 5 (male, 40s) asked: “In the COVID situation, it has not changed. Like, what is my life? It is not changed much. Because the way they treat me in 2020, is what a treatment in 2019 is, or 2018.” This lack of change in NHS services and understanding of SCD by healthcare professionals, as well as triaging, heightened fears of chronicity and mortality.

### Mortality and chronicity

While time seemed to slow down during the pandemic, some participants noted a speeding-up of time or blurring of time because days were always the same. Crip-time is not always ‘slow’ but can, due to the way in which mind and body interact, speed up (Price, 2015), or collapse completely into loss of time in a pain crisis. Understandings of time became connected to the emotions, such as anxieties and fears correlated to why they had to shield and what they heard was happening in clinical settings to older and disabled people who did get COVID-19, as found in the literature (Monahan et al., 2020; Shakespeare et al., 2021; Ramirez et al., 2022). Participant 8 (female, 20s) explained:

“It's crazy when you think about time and I feel like this last year has just been a whirlwind (…) I was thinking about this and I think it's what has my experience of shielding been like and I think it's been really varied. So right at the start, I was really scared, I think I think we saw 12 weeks. And I was like, What? That's three months not going anywhere else. I don't think I could do that. I think just the fear, you heard all these things on the news saying that, actually, they're going to be rationing ICU and oxygen.”

Many participants found ‘triaging’ and disablism in services very emotionally triggering, but it was only when both participants 8 and 6 explained how their mothers had watched over them and protected them in NHS services that we understood why. Participant 6 (female, 20s), when recounting how she was watched over in an intensive care unit, recounted:

“In fact he was always telling my mom, we've used all our equipment, you know, basically he's saying we're wasting our product, there’s no brain activity, no nothing, nothing is working, if she were to wake-up, you would wish she was dead because she is not going to be able to be on her own, forever, she's just going be in a wheelchair for the rest of her life. So why would you want to make her be in that result? You know she's independent, stuff like that.”

The above quote illustrates disablism and ableism in healthcare services and gives credence to the fears and anxieties that participants had linked to going into the hospital during the pandemic with nobody to watch over them as adults. Heightening the fears even more was the lack of control that they had over the severity of a pain crisis. Participant 8 (female, 20s) explained chronicity as ongoing:

“I live with a chronic illness (…) I cannot tell you after this crisis this is the last crisis I am ever going to have my life. That is a lie. Even if I tried to say that, it would be lie because I will have a crisis again, you will, sadly have a crisis again, whether we like it or not, that is part of the disease.”

Furthermore, participants also noted that for them, the intersectionality of the ethnicity of healthcare professionals who were at greater risk was ‘unsettling’ as they were from the same minority ethnic background. So, participant 9 (female, 30s) stated:

“And I think when it showed that the healthcare workers that were dying, they were either Black or Asian, you know. It was very worrying, very worrying, very upsetting because you just think what’s happening and what is the reasons for all of that? And it just seemed like all we knew was that you’re more at risk but no one was really explaining why?”

The lack of information was also distressing because most of the participants had lost family members or knew people who had died during the pandemic. The risks of death were correlated to inequalities and racism in broader society, and several of the participants made links to the Black Lives Matter Movement (Sobo et al., 2020). Participant 3 (male, 60s) noted that it was nothing new:

“So, there's a lot things that I experienced, you know what I mean but like I says, that's why I felt it when George Floyd got killed. I turned off the news feed. I didn't want to know because until you until you know or identify the brutality, discrimination, inequality, the harrassment as a Black male …”

The issues of racism in the NHS were often connected to pain management and treatment because they intersected with racist stereotypes as well as ableism in the invisibility of pain, which was contested (Berghs et al., 2022). Participant 6 (female, 20s) noted how she was viewed as a drug addict and told by a nurse, “Oh, you have just got a habit.” Similarly, participant 8 (female, 20s) noted that when seriously in danger of death and disablement, a nurse told her, “Oh, you could look a bit more ill.” Participants explained how difficult it was to know what to do in such situations. Noteworthy, is that the older male participants felt that they either had to acquiesce and become ‘smaller” or ‘passive’ while the younger female participants wanted to fight for better treatment but then became labelled as ‘aggressive’. Yet, participant 2 (female, 20s) explained how it was important to talk about racism but also how to know when to ‘give time’ mattered:

“I'm adding to the problem. If I don't say anything, or if I don't question it, or try and get out of that mindset, I just have to accept it, then nothing will ever change. So even I'm learning how to have these conversations. And like, think of me now, what I feel like is racist or figuring out who am I going give the time to educate? And who am I just going to ignore? Like, it's kind of learning on both sides?”

However, she also noted that there would be times when she could not ‘give’ that time. As such, participant 2 said it was important to always have a family member or advocate present when arguing:

“Even though, especially this is more for adult care, even though we are adults from ages 16 and that sort of transition, even if we're 35, or 40, whatever. And we're in a crisis we're like extremely vulnerable, like we can't talk about ourselves. So, the same way that you would give an older person, like they use like this assessment called DoLS, which is like they assess, if that person can make decisions for themselves in the present moment, things like that, we should have that same assessment.”

We did not expect any correlations to be made to age and ageing, but the highlighting of the ages of 35–40 was significant in this participant’s account as she identifies the vulnerabilities people with SCD experience at that age, like older and disabled people. We also noted that the older participants brought up the greater risks of co-morbidities and early ageing. Many also had additional needs for medical care for co-morbidities, such as diabetes, which they argued healthcare professionals seemed unaware of. This increased the risk of diagnostic overshadowing with no clear understanding of the boundaries between age and impairment effects (van der Horst and Vickerstaff, 2022). We also noted that participants understood this early ageing and could explain how it impacted impairments, like participant 5 (male, 40s):

“We have problems (…) getting worse and worse and worse. Because now I use glasses just to read or to (…) But I know like, people with SS, we have more, like the problems with the lungs, with the eyes, with the bones and stuff.”

Living with a chronic condition also has a mental health connection to understanding the impact of early ageing as participants explained how the pandemic heightened fears of risks and thus death. Participant 4 (female, 30s) explained why she suffered from anxiety:

“When I was younger, I was quite healthy, to be fair, but when I would fall sick, it would get me down. Because there were people who were my age, and I felt that I should be doing what they were doing. So, I think that’s something that professionals need to take into consideration. And then when I had my (child), my anxiety really went sky high, because then all I was thinking about was my mortality to be to be fair, because if I had a crisis, and I passed away, who would look after (them), and I think it's things that people who don't have an illness like we do, don't think about at this age, but it's something that we do think about.”

All the participants explained that regardless of their physical health, they had mental health needs that were not being met and that were poorly understood. The pandemic also brought out a need for bereavement support, which some of the youngest participants, 2 and 6, felt had been ignored and needed a culturally sensitive response. Participant 6 (female, 20s) said:

There needs to be some form of grief counselling as well for the other patients that have sickle cell because we are really close knit and we all depend on each other and the majority of us was known to each other (…) some of us will be in hospital when another person with sickle cell passes away. So, it's sad for that person and their family. But then you as a person with sickle cell think, is that going to be me? Like that person was only 30? Is that what is going to happen to me when I'm 30 but nobody talks about it. So, I think there needs to be like a recognition that there needs to be some kind of grief counselling.

Noteworthy are the connections being made to the death of a person with SCD at age 30, the heightening of fears of mortality, and the lack of recognition of grief and the need for psychological support. All participants noted how they were living with ‘chronicity’, a chronic illness that was becoming progressively more complex and uncertain (Rouse, 2009), which had a mental health impact. It could also at any time become acute and life-threatening, the fears of which the pandemic was amplifying.

## Discussion

In the findings, there are commonalities to many of the threads woven in the literature on the pandemic and how it impacted people identified as CEV, like the creation of vulnerabilities in older and disabled populations and the lack of disability and culturally inclusive services (Shakespeare et al., 2021). However, people with SCD experienced intersectionality of discriminations (Crenshaw, 1991) and clearly used words like racism and oppression to make sense of their experiences during shielding, within NHS services, and also in British society. We found that multiple discriminations were understood and highlighted through concrete examples, even if participants did not necessarily use words like ableism (Campbell, 2008, 2009) or disablism (Oliver, 1986). They understood the connections to disability and the lack of human rights being afforded to people like themselves. For instance, the inhumanity of triaging, the hierarchy of illnesses, and the prioritisation of some conditions over others in hospitals were all mentioned (Abrams and Abbott, 2020). Similarly, while nobody mentioned necropolitics (Thorneycroft and Asquith, 2021), some participants did note the loss of lives of ethnic minority people and disabled people due to multiple discriminations that can also impact people with SCD. While most disability models see disability as a central experience and the removal of barriers leading to emancipation (Oliver, 1983, 2013), we found that disability was one of many discriminations that people could experience. This may point to needs within disability politics and models for a greater conceptual understanding of intersectionality (Crenshaw, 1991) to ensure inclusion.

While we also found that people with SCD had worse mental health during the pandemic, there were nuances in that some people reported better physical health and resilience in coping, similar to Kemp et al. (2020). We did not find a decrease in mental health needs mid-pandemic (Robinson et al., 2022), but this might have been due to the longer periods of shielding and lockdowns that people in some parts of the Midlands experienced. We did find that anxieties and fears were connected to the multiple discriminations that people noted during the pandemic, which were heightened due to people’s inability to properly ‘shield’ but also because there was a lack of psychological support in services. We noted there was some evidence of ‘weathering’ traumas in hospital settings in the examples given by participants, which were also correlated to growing up with a chronic condition and experiences of structural racism (Geronimus, 2023). However, we could not assess if this had an embodied impact or not in accelerated ageing which then impacted on SCD, or if it was SCD that then impacted the body. We also struggled with how to conceptualise the embodied impact of weathering (Geronimus, 2023), when it was possible that it was caused by multiple discriminations across the life course as well as disadvantage. We found there were discriminations based on invisible forms of early ageing that perhaps services were unaware of, which participant 4 understood as affecting all the organs in his body. Was this an accelerated third or fourth part of life typically seen in later life ageing and disablement (Gilleard and Higgs, 2011) or a second transition we should prepare for in services? Our participants noted that ageing and death could happen earlier for people with SCD, but it was not clear if we could separate ‘age effects’ from ‘impairment effects’ as van der Horst and Vickerstaff (2022) argued. Another issue was how to conceptualise a pain crisis that could act as both acute and chronic. This revealed limitations within models of disability that tried to separate impairment from disablement (Oliver, 1983, 2013), as a pain crisis could severely disable but also lead to death. This could happen in the best-case scenario, where all medical assistance and support were provided. Similarly, a person could also have a severely disabling condition when younger that became manageable as chronic as they aged, meaning a lessening impairment and, thus, progressively less disabling.

None of our participants mentioned ‘crip-time’ (Kafer, 2013), which we did not expect, but they seemed to have an implicit understanding of it by living with differing forms of chronicity. All participants explained that to prevent impairment effects and serious pain crises, timing life events and understanding an embodied temporality (connection between mind and body in body–mind; Price, 2015) were critical to living life well (Sheppard, 2020; Samuels and Freeman, 2021). The older that our participants became, the more they understood how their environment and emotional states were linked to their physical and mental health, as well as differing SCD physical, sensory, and cognitive symptoms or impairments and age effects (van der Horst and Vickerstaff, 2022). It was often our older participants who explained how time was linked to caring for oneself and others, for instance, to plan time to be well with family, to rest, or that time was needed to guard good health and anticipate a pain crisis. Participants also related living in the moment, and participant 3 correlated this understanding of time to his Christian beliefs.

Participants were thus expressing an embodied ethics correlated to how to live well with SCD and cope with periods of acute crisis. Akin to Bailey’s (2021) conception of ‘ethics of pace’, they noted the ethical and existential need to ‘pace’ oneself in life to protect their wellbeing. This is different from the ‘pace’ that Sheppard (2020: 45) describes in rehabilitative services, which is about ascribing to a ‘normative way of moving through time’ ending with an ‘inevitable failure to do so’ and is also more than non-normative self-care. Tremain (2023) argues that we now need an ontology and epistemology of crisis, but this already exists for people with SCD. As participant 8 states, “I will have a crisis again, you will, sadly have a crisis again, whether we like it or not, that is part of the disease.” They explain that this is ‘inevitable’ but they are ‘more’ than their pain. In statements like this, an ‘ethics of crisis’ is embodied in values and norms correlated to temporality and notions of the self as body–mind emerge. Participants normatively learn how to physically and psychologically exert control over their environment, ensure support, and cope with an uncertain condition. They also noted times when they would be ‘on the floor’ or be incapacitated during a pain crisis, like a disabled and/or older person, exposing them to the risks of unethical treatment and care, not only to racism but also to ableism, disablism, and ageism. They understood these limits and that they had no control over a pain crisis or even mortality, but they had embodied ethics of how to live with the inevitability of crises before the pandemic. Both impairment and age effects are invisible and can be contested (van der Horst and Vickerstaff, 2022), but they are also masked by one impairment effect (pain) over all others. Just as a person with SCD goes into crisis and loses control over the body and time, the pandemic and indignities of shielding as a CEV person meant a loss of control of embodied vulnerabilities and temporality, heightening individual fears of early ageing, disablement, and early mortality that society and services were contributing to.

## Conclusion

People with SCD understand that they will have times in their lives with a worse quality of life and periods when they need care and support. They explained how a relational, embodied ethics of crisis was helpful to manage the chronicity and medicalisation of SCD. They, and their families, had developed ways and embodied expertise to cope with and temporarily manage the condition. This ability to adapt to SCD had also been formed within a society and NHS services where they experienced structural racism as well as discriminations like ableism, disablism, and even ageism. This meant that the ethics of crisis management became embodied, hypervigilant, and incorporated into a necessary pace of caution and anticipation. Anticipation of crisis of pain, which was also a crisis of time in that it meant a total loss of temporality and self. The pandemic as a crisis writ large had a psychological impact, which our participants related to as affecting them and their families and which became heightened and linked to fears of early mortality.

## Data availability statement

The datasets presented in this article are not readily available because due to the sensitive nature of the discussions around health, morality and racism we have restricted access to the data to only team members. Requests to access the datasets should be directed to Maria.Berghs@dmu.ac.uk.

## Ethics statement

The studies involving humans were approved by the De Montfort University Faculty of Health and Allied Health Sciences Ethics Committee. The studies were conducted in accordance with the local legislation and institutional requirements. The participants provided their written and verbal consent informed consent to participate in this study.

## Author contributions

MB: Conceptualization, Data curation, Formal analysis, Funding acquisition, Methodology, Project administration, Resources, Writing – original draft, Writing – review & editing, Investigation, Software, Supervision, Validation. FH: Conceptualization, Data curation, Formal analysis, Methodology, Software, Writing – original draft, Writing – review & editing. SY: Conceptualization, Formal analysis, Investigation, Methodology, Writing – original draft, Writing – review & editing. RK: Conceptualization, Data curation, Formal analysis, Investigation, Methodology, Project administration, Writing – original draft. AW: Conceptualization, Data curation, Formal analysis, Funding acquisition, Investigation, Methodology, Project administration, Supervision, Writing – original draft.
